# Can spatial sorting associated with spawning migration explain evolution of body size and vertebral number in *Anguilla* eels?

**DOI:** 10.1002/ece3.2671

**Published:** 2016-12-25

**Authors:** Anders Forsman, Hanna Berggren

**Affiliations:** ^1^ Ecology and Evolution in Microbial Model SystemsEEMiSDepartment of Biology and Environmental ScienceLinnaeus UniversityKalmarSweden

**Keywords:** *Anguilla*, body size, dispersal, evolution, fish, pleomerism, spawning migration, vertebrae

## Abstract

Spatial sorting is a process that can contribute to microevolutionary change by assembling phenotypes through space, owing to nonrandom dispersal. Here we first build upon and develop the “neutral” version of the spatial sorting hypothesis by arguing that in systems that are not characterized by repeated range expansions, the evolutionary effects of variation in dispersal capacity and assortative mating might not be independent of but interact with natural selection. In addition to generating assortative mating, variation in dispersal capacity together with spatial and temporal variation in quality of spawning area is likely to influence both reproductive success and survival of spawning migrating individuals, and this will contribute to the evolution of dispersal‐enhancing traits. Next, we use a comparative approach to examine whether differences in spawning migration distance among 18 species of freshwater *Anguilla* eels have evolved in tandem with two dispersal‐favoring traits. In our analyses, we use information on spawning migration distance, body length, and vertebral number that was obtained from the literature, and a published whole mitochondrial DNA‐based phylogeny. Results from comparative analysis of independent contrasts showed that macroevolutionary shifts in body length throughout the phylogeny have been associated with concomitant shifts in spawning migration. Shifts in migration distance were not associated with shifts in number of vertebrae. These findings are consistent with the hypothesis that spatial sorting has contributed to the evolution of more elongated bodies in species with longer spawning migration distances, or resulted in evolution of longer migration distances in species with larger body size. This novel demonstration is important in that it expands the list of ecological settings and hierarchical levels of biological organization for which the spatial sorting hypothesis seems to have predictive power.

## 1 Introduction

There is a growing awareness that to better understand the drivers influencing biological diversity, it is necessary to consider the extent and nature of nonrandom, genotype‐dependent dispersal (Berggren, Tinnert, & Forsman, 2012; Bolnick & Otto, 2013; Edelaar & Bolnick, 2012; Edelaar, Siepielski, & Clobert, 2008; Phillips, Brown, Webb, & Shine, 2006; Reznick & Ghalambor, 2001; Shine, Brown, & Phillips, 2011). The proposition (Lomolino, 1984; Shine et al., 2011) that evolutionary change might be driven by spatial sorting in the form of phenotype‐ and habitat‐dependent dispersal capacity is particularly interesting in this context.

Spatial sorting, sensu Shine et al. (2011), is a process that can contribute to evolutionary change by assembling phenotypes and genotypes through space rather than through time, with variation in the capacity for dispersal being the fundamental driving force. Spatial sorting may result in an overrepresentation of dispersal phenotypes at invasion fronts and geographic range margins, where it can subsequently give rise to assortative mating. Given that the dispersal‐favoring traits are inherited, this can result in the production of offspring with even more extreme dispersal trait values in the next generation (Berggren et al., 2012; Shine et al., 2011). Spatial sorting may thus promote rapid evolutionary change between generations even if the dispersal‐favoring trait does not directly influence reproductive success (Shine et al., 2011).

**Figure 1:**
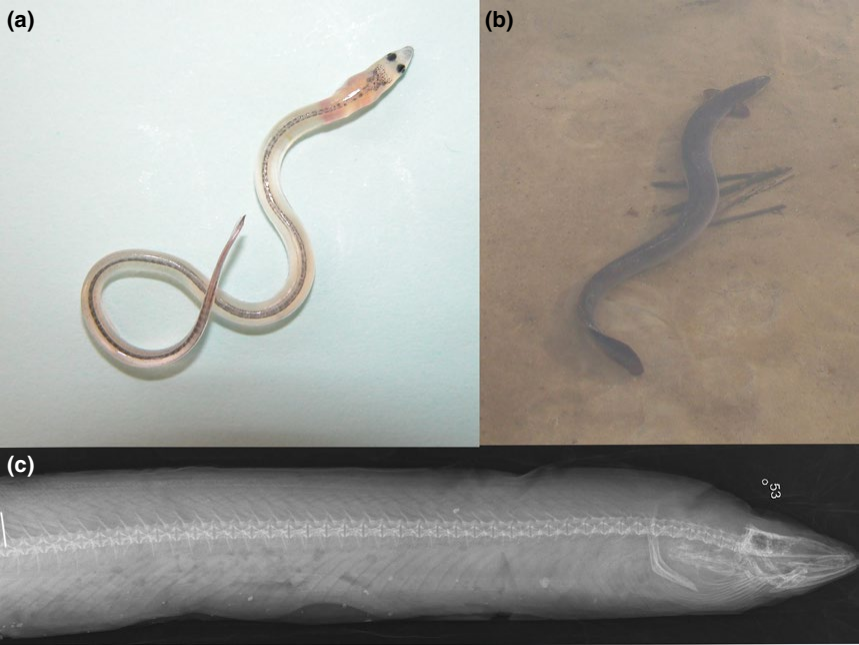
The European eel *Anguilla anguilla*. (a) A juvenile “glass eel.” Photograph: Håkan Wickström. (b) An adult eel, 87 cm long, 1,360 g. Photograph: Oscar Nordahl. (c) X‐ray showing the anterior part of the vertebral column and head of an adult eel. Photograph: H. Berggren

The key pattern which the spatial sorting hypothesis was put forward to explain concerns an overrepresentation of *Rhinella marina* cane toads with longer legs (compared with their body size) at the rapidly expanding invasion front in northern Australia (Phillips et al., 2006; Shine et al., 2011). Spatial sorting has been suggested to play a role also in other species and for the evolution of different types of traits. For instance, populations of wing polymorphic Tetrigidae pygmy grasshoppers that inhabit newly colonized, disturbed environments have a higher incidence of the macropterous long‐winged, flight‐capable phenotype (Berggren et al., 2012). Insular populations of *Microtus agrestis* field voles that inhabit more isolated and fragmented archipelagos and are subject to rapid extinction–recolonization dynamics have larger body sizes and longer hind feet (Forsman, Merilä, & Ebenhard, 2011). Differential results from comparisons among populations of morphological traits related to dispersal and traits related to foraging point to a role of spatial sorting in facilitating the expansion of the common myna *Acridotheris tristis* in South Africa (Berthouly‐Salazar, van Rensburg, Le Roux, van Vuuren, & Hui, 2012). All these previous examples of evolution that might have been driven by spatial sorting represent within species studies building either on spatial comparisons among populations inhabiting different environments, or on longitudinal approaches and demonstrations of changes in dispersal‐favoring traits over time within populations. To our knowledge, the spatial sorting hypothesis has hitherto not been evaluated using comparisons across species. We also are not aware of any previous attempt to specifically evaluate the spatial sorting hypothesis in fishes. Freshwater eels belonging to the genus *Anguilla* (Figure 1) provide a good model system to fill these gaps and add a layer of generality to this issue.

In its “neutral” form, cumulative spatial sorting and the resulting assortative mating process can potentially drive the evolution of more dispersive phenotypes even if variation in the dispersal‐enhancing traits is not associated with lifetime reproductive success, that is in the absence of traditional natural selection (Phillips et al., 2006; Shine et al., 2011). Shine et al. (2011) state that the effects of spatial sorting can be distinguished from effects of classical natural selection because the latter predicts fitness to be uncorrelated or negatively correlated with dispersal rate at invasion fronts. However, the proposition to empirically evaluate the spatial sorting hypothesis in this manner comes with a philosophical dilemma. Nonsignificant associations between fitness and dispersal capacity would not provide evidence in support of “neutral” spatial sorting because statistical tests have been designed to reject (not confirm) null hypotheses of *no* association or no difference. Of course, it does not follow from this general problem with directional null hypothesis that neutral spatial sorting cannot be an important driver of evolution of biological diversity, but it does complicate demonstrating its role. In many systems, spatial sorting and assortative mating may not operate alone but in interaction with traditional selective forces that also act on and mold the evolution of dispersal traits affecting endurance or speed. With a less restrictive view, the spatial sorting process is not only relevant for range‐expanding systems in which sorting operates in a repeated manner. Accepting that spatial and/or temporal sorting associated with variation in dispersal capacity may operate together with classical natural selection broadens the conditions and type of biological systems for which the spatial sorting process may have predictive power. Here, we report on a comparative study of freshwater *Anguilla* eels as an empirical example of the rationale.

*Anguilla* eels are catadromous, meaning that they spawn in the sea and feed and grow in freshwater areas (Aoyama, 2009; Aoyama & Tsukamoto, 1997; Ege, 1939; Silfvergrip, 2009; Tesch, 2003). Depending on species, they remain in freshwater habitats for 15–30 years before they return to the open sea and initiate the migration back to the spawning areas where they were once born. Freshwater eels attract much scientific attention, have socioeconomic importance, and are generally being subject to conservation concerns. Of the 13 *Anguilla* species included in the IUCN Red List of Threatened Species (IUCN 2015), eight species are listed as critically endangered, endangered, vulnerable, or near threatened, two species have been assigned to the group least concerned, and three species are classified as data deficient. The freshwater eels are famous for their remarkably long spawning migrations (Amilhat et al., 2016; Beguer‐Pon, Castonguay, Shan, Benchetrit, & Dodson, 2015; Righton et al., 2016), and there is considerable variation among *Anguilla* species in the distance between the spawning site and the areas used for growth (Aoyama & Tsukamoto, 1997). Eels have an elongated snake‐like body shape, and there is variation both within and among species in two dispersal‐favoring traits, namely body length and vertebral numbers (Ege, 1939; Silfvergrip, 2009; Tesch, 2003) (Figure 1, Table 1).

**Table 1:** Data on maximum body length and vertebral numbers for 18 species of *Anguilla* eels

Species	Maximum body length (cm)	Total vertebral number mean (max)	Abdominal vertebral number mean (max)	Caudal vertebral number mean (max)
*A. anguilla*	150^a^	114.7 (119)^a^	45.2 (47)^a^	69.5 (72)^a^
*A. australis australis*	130^c^	112.6 (116)^b^	46.2 (48)^b^	66.4 (68)^b^
*A. bengalensis bengalensis*	120^a^	109.1 (112)^b^	40.7 (42)^b^	68.4 (70)^b^
*A. bicolor bicolor*	120^c^	109.5 (115)^b^	43.3 (45)^b^	66.2 (70)^b^
*A. borneensis*	80^a^	105.5 (108)^a^	40.6 (42)^a^	64.9 (66)^a^
*A. celebesensis*	60^a^	103.3 (107)^a^	39.0 (41)^a^	64.3 (66)^a^
*A. dieffenbachii*	200^a^	112.7 (116)^a^	44.3 (47)^a^	68.4 (69)^a^
*A. interioris*	80^a^	104.4 (107)^a^	40.3 (42)^a^	64.1 (65)^a^
*A. japonica*	130^a^	115.8 (119)^a^	43.6 (45)^a^	72.2 (74)^a^
*A. bengalensis labiata*	145^a^	111.3 (115)^b^	40.9 (42)^b^	70.3 (73)^b^
*A. marmorata*	200^a^	105.6 (110)^a^	41.1 (43)^a^	64.5 (67)^a^
*A. megastoma*	90^a^	112.7 (116)^a^	41.7 (44)^a^	71.0 (72)^a^
*A. mossambica*	150^a^	102.9 (106)^a^	40.5 (42)^a^	62.4 (64)^a^
*A. obscura*	110^a^	104.0 (108)^a^	41.5 (43)^a^	65.5 (65)^a^
*A. bicolor pacifica*	123^c^	107.1 (111)^b^	43.1 (45)^b^	64.0 (66)^b^
*A. reinhardtii*	165^a^	107.8 (110)^a^	42.6 (44)^a^	65.2 (66)^a^
*A. rostrata*	152^a^	107.2 (111)^a^	42.8 (45)^a^	64.4 (66)^a^
*A. australis schmidtii*	100^d^	111.7 (115)^b^	45.7 (48)^b^	66.0 (67)^b^

Key to references: ^a^Silfvergrip (2009); ^b^Ege (1939); ^c^FishBase (2015); ^d^Retrieved from http://www.Nationalaquarium.co.nz

Previous studies suggest that larger body size is linked to increased locomotion, endurance, and a higher capacity for long‐distance dispersal in various organisms (Forsman, 1996; Forsman et al., 2011; Foster, 1964; Hemptinne, Magro, Evans, & Dixon, 2012; Jenkins et al., 2007; Lawlor, 1982; Lomolino, 1984, 1985; McDowall, Mitchell, & Brothers, 1994; Roff & Fairbairn, 2001). Several lines of evidence indicate that also vertebral number (henceforth VN) is associated with maneuverability and speed of locomotion (Arnold, 1988; Arnold & Bennett, 1988; Kelley, Arnold, & Glatstone, 1997; Long et al., 2011; McDowall, 2003; Swain, 1992a; Webb, 1975). For instance, VN influences body flexibility and the ability to curve the body (Ackerly & Ward, 2016; Brainerd & Patek, 1998). Given that VN influences swimming performance, it is reasonable to hypothesize that VN impacts on the capacity for long‐distance migration and the aptitude to cope with physical obstacles faced during migration (Kelley et al., 1997).

**Figure 2:**
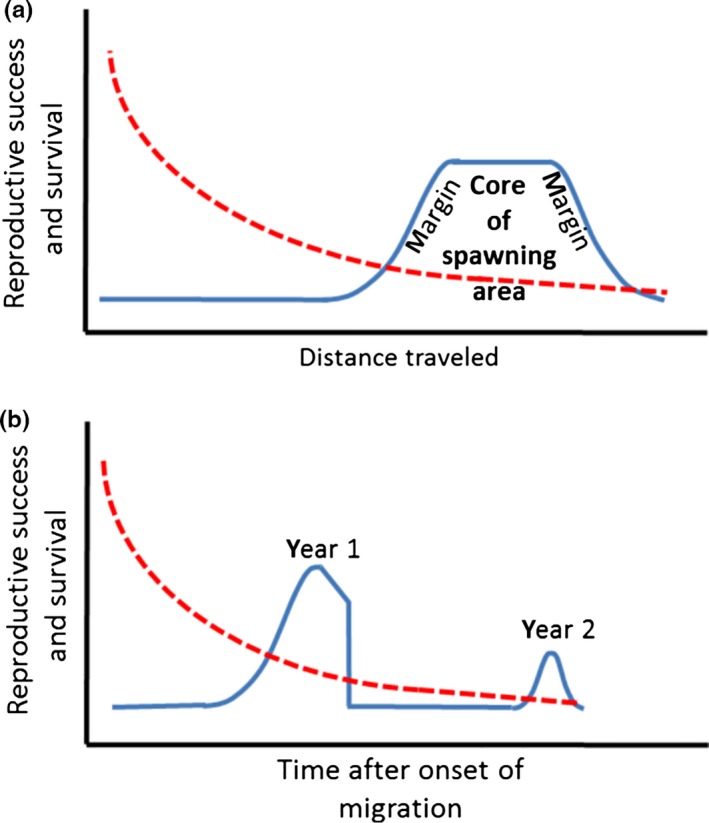
Graphical model illustrating how variation in dispersal capacity might generate assortative mating and together with (a) spatial variation in quality of spawning area and (b) temporal variation in quality of the spawning area influence both reproductive success and survival of spawning migrating eels. Reproductive success (blue lines) is assumed zero for individuals that do not reach the spawning area at all or do not arrive in time and then increases asymptotically as habitat quality increases from marginal to core areas (a) or from early to late in the spawning season (b). Survival (red hatched lines) declines with distance traveled (a) and with time after onset of migration (b) owing to predation risks and energetic costs associated with migration. The lower peak in reproductive success in the bottom panel represents individuals that arrive too late to the spawning area and postpone reproduction to the second year (Righton et al., 2016), such that energy reserves are further depleted

Because of the challenges associated with long‐distance migration, traditional natural selection probably acts against eel individuals that do not make it to the spawning area at all, or not in time (Figure 2). Results from a recent study of migration behavior of European eels that were electronically tagged indicate that mortality during spawning migration is very high; approximately half of those tagged individuals that migrated for longer than one week (*n *=* *41) suffered predation (Righton et al., 2016). Such high mortality is likely to favor individuals possessing dispersal‐enhancing traits and drive the evolution of genetic and phenotypic features linked to dispersal capacity via traditional natural selection, the result of which is likely amplified by the sorting process. In addition, there is potential for assortative mating mediated via spatial and temporal sorting owing to differential dispersal capacity to further influence evolution in this type of system.

If large body size and high VN promote long‐distance dispersal in eels, then there are at least two ways by which this may generate assortative mating in space or time. First, if eels arrive from various directions, then it is possible that good dispersers postpone reproduction until they have reached the core of the spawning area and that individuals with a poorer capacity for dispersal initiate reproduction at the outskirts of the spawning area to reduce migration distance, such that dispersal phenotypes are separated in space. If eels come from one direction only, there might instead be a gradient not from edge to core but from close to far from the mainland. Second, the arrival of fast and slow swimmers to the spawning area might be separated in time, especially if individuals initiate their spawning migration at about the same time.

Based on the above reasoning, the spatial sorting hypothesis (Shine et al., 2011) predicts that there should be assortative mating with regard to dispersal‐enhancing traits in space and/or time. If the classical selective forces are absent, spatial or temporal assortative mating might increase the variance of dispersal‐enhancing traits (assuming the traits are heritable), but not contribute to any change of mean trait values. If there are habitat quality differences between core and marginal areas of the spawning area, or between areas close or far from the mainland, then this could drive directional evolutionary change, with spatial sorting being a key element (Figure 2a). Similarly, the potential for temporal assortative mating to drive directional evolutionary change increases if the quality of the spawning area declines over time, as in the case of the European eel (Righton et al., 2016; Figure 2b). These proposed scenarios should have resulted in the evolution of longer bodies and larger vertebral numbers in species of eels having longer spawning migration distances.

To evaluate these predictions, we compile data on body length, vertebral number, and spawning migration distance for 18 species (three subspecies) of *Anguilla* freshwater eels. Next, we search for associations across species between the three types of phenotypic traits. However, species that share an evolutionary history cannot be considered as statistically independent observations (Felsenstein, 1985; Garland, Harvey, & Ives, 1992; Harvey & Pagel, 1991; Purvis & Rambaut, 1995). We therefore take advantage of a phylogenetic hypothesis for *Anguilla* species (Minegishi et al., 2005) and use a comparative approach based on analysis of phylogenetic independent contrasts (Felsenstein, 1985; Purvis & Rambaut, 1995) to evaluate whether evolutionary shifts in spawning migration distance have been accompanied by evolutionary shifts in body size or in VN.

## 2 Materials and Methods

### 2.1 Data on body length, vertebral numbers, and migration distances

Information on maximum body length and VN was obtained from the literature (Ege, 1939; FishBase 2015; Silfvergrip, 2009; Table 1). Eels have indeterminate growth. We therefore used information on maximum body length, to reduce the risk that our analyses of and results for body size be heavily biased by differences in age structure or any inconsistency with regard to the inclusion or exclusion of subadult individuals among samples for the different species.

The vertebral column of fish can be divided into an abdominal and a caudal region with partly different functions (Tesch, 2003). The abdominal region is primarily linked to feeding, digestion, and reproductive capacity, whereas the caudal region is of particular importance for propulsion and swimming capacity. Accordingly, results from previous comparative analyses of eels and other elongated fishes suggest that selection on and evolutionary shifts in abdominal and caudal VN may be independent (Mehta, Ward, Alfaro, & Wainwright, 2010; Ward & Brainerd, 2007). We therefore collected data on total, caudal, and abdominal VNs. Maximum and mean VNs were strongly correlated across species (total VN: *r *=* *.984; abdominal VN: *r *=* *.985; caudal VN: *r *=* *.963, all *n *=* *18, all *p *<* *.0001).

Information on geographic distributions and location of spawning areas was compiled from the literature (Aoyama, 2009; Aoyama, Wouthuyzen, Miller, Inagaki, & Tsukamoto, 2003; Tesch, 2003; Tsukamoto et al., 2011). This information was used for estimating maximum migration distance to spawning area for each species, by measuring the distance between the spawning site and the endmost point of the distribution area, using computer software Google Earth 6.0 (see Table S1).

We first investigated whether caudal and abdominal VN was positively associated across species, and whether evolutionary shifts (increments and decrements) in the two traits have been correlated. We next tested for an association of total VN with maximum body length. Vertebral number may influence the capacity for growth because individuals with more vertebrae have more growth zones (Harding, 1985; Lindell, Forsman, & Merilä, 1993). Because VN affects locomotion and agility, it may also influence foraging performance (Arnold, 1988; Swain, 1992b; a; Lindell et al., 1993; Tibblin, Berggren, Nordahl, Larsson, & Forsman, 2016). Previous studies based on among‐species comparisons indicate that the evolution of a larger and more elongated body shape has been accompanied by evolution of increasing VN in both snakes (Lindell, 1994) and fish (Lindsey, 1975; Maxwell & Wilson, 2013). Our aim was not to specifically test for such pleomerism. The most important analyses in our study with regard to the spatial sorting hypothesis concerned the associations of body length and VN with migration distance.

### 2.2 Statistical analyses

We first tested for associations across species between body length, VN, and spawning migration distance using Pearson correlation analyses. In these “tips analyses,” species were treated as independent observations.

Next, we calculated phylogenetic independent contrasts, following the approach put forward by Felsenstein (1985). For this, we used computer program CAIC (comparative analysis by independent contrasts, version 2.6.8.b; Purvis & Rambaut, 1995). This phylogeny‐based approach allows for reconstruction of evolutionary transitions of trait values, and for evaluation of the null hypothesis that traits have evolved independently of each other against the alternative hypothesis that changes have been correlated throughout the phylogeny (Felsenstein, 1985; Garland et al., 1992; Harvey & Pagel, 1991). Because our data only included continuous variables, we used the crunch algorithm (Purvis & Rambaut, 1995). The tree topology and branch lengths used in the analyses were derived from published phylogenetic analyses of the genus *Anguilla* based on the whole mitochondrial genome (Minegishi et al., 2005). Data on body length, VN, and migration distances were log‐transformed prior to analyses of independent contrasts (Purvis & Rambaut, 1995). The CAIC approach builds upon Felsenstein's model according to which evolution of continuous characters can be modeled as a random walk process (Felsenstein, 1985; Purvis & Rambaut, 1995). This in itself provides a strong reason for logarithmic transformation of data in comparative analyses. As pointed out by Purvis and Rambaut (1995), an increase in size of one kilogram is much more likely in a whale lineage than in a lineage of shrews, and log transformation of size data makes the reasonable assumption that different lineages are equally likely to make the same proportional (rather than absolute) evolutionary change in size.

Because the hypotheses under investigation were directional (e.g., dispersal promotes evolution of longer bodies and/or larger VNs—we had no reason to hypothesize that increments in migration distance should be associated with decrements in body length or VN), statistical significance was assessed using one‐tailed tests (Rice & Gaines, 1994).

Results from the analyses described above showed that abdominal and caudal VNs were positively correlated (see Results). When testing for associations with migration distance, we therefore used data on total VN, rather than testing for an association of migration distance with two different traits that were not independent. Results below are based on analyses of data on maximum VN, unless otherwise stated.

## 3 Results

There was considerable variation among the 18 *Anguilla* species in maximum body lengths (range 60–200 cm), total VN (range 106–119), abdominal VN (range 41–48), caudal VN (range 64–74), and spawning migration distances (range 750–8,200 km, Table 1).

**Figure 3:**
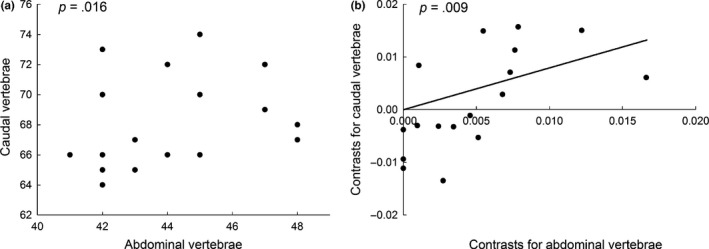
Relationship between number of vertebrae in the caudal and abdominal regions in *Anguilla* eels, (a) as evidenced by associations across species and (b) as evidenced by evolutionary shifts estimated using phylogenetic independent contrasts. *p*‐Values indicate one‐tailed probabilities associated with correlation analysis of raw data (a) and regression analysis of independent contrasts through the origin (b). The line (b) indicates the regression line

Abdominal VN was not correlated with caudal VN across species (results from the tips analysis, *r *=* *.25, *p *=* *.16, *n *=* *18, Figure 3a). However, independent contrasts analysis indicated that evolutionary shifts in the number of abdominal vertebrae have been significantly correlated with shifts in the number of caudal vertebrae (*F*_1, 16_ = 6.65, *p *=* *.0101, Figure 3b). The result regarding correlated evolution was similar when data for mean rather than maximum VN were used (*F*_1, 16_ = 6.95, *p *=* *.009).

**Figure 4:**
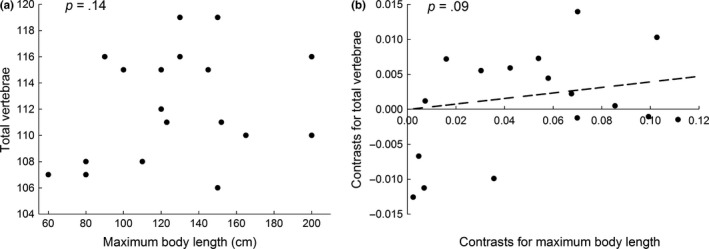
Relationship between total number of vertebrae and body length in *Anguilla* eels, (a) as evidenced by associations across species and (b) as evidenced by evolutionary shifts estimated using phylogenetic independent contrasts. *p*‐Values indicate one‐tailed probabilities associated with correlation analysis of raw data (a) and regression analysis of independent contrasts through the origin (b). The dashed line (b) indicates the regression line

Body length was not correlated with total VN across species (*r *=* *.26, *p *=* *.145, *n *=* *18, Figure 4a). Evolutionary shifts in body length were not associated with evolutionary shifts in total VN (*F*_1, 16_ = 1.97, *p *=* *.09, Figure 4b).

**Figure 5:**
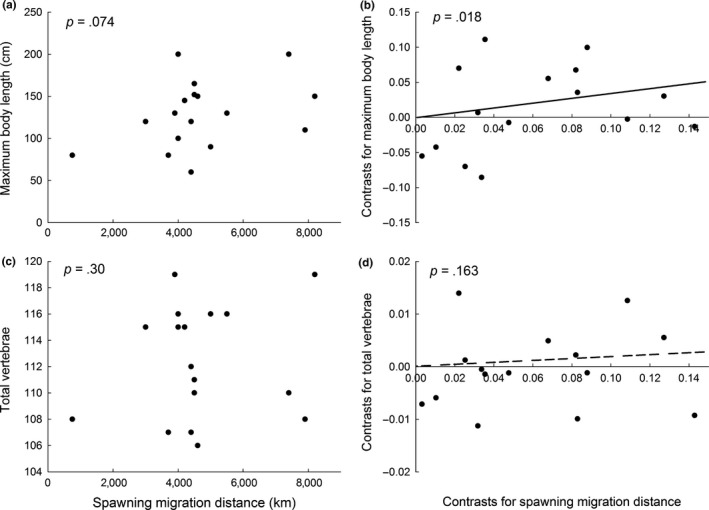
Associations of spawning migration distance in *Anguilla* eels with (a and b) maximum body length and with (c and d) total number of vertebrae. Relationships represent associations based on (a and c) cross‐species comparisons and (b and d) evolutionary shifts as estimated using phylogenetic independent contrasts. *p*‐Values indicate one‐tailed probabilities associated with correlation analysis of raw data (a and c) and regression analysis of independent contrasts through the origin (b and d). The line (b) and dashed line (d) indicate regression lines

Body length tended to increase with increasing spawning migration distance across species (*r *=* *.37, *p *=* *.074, *n *=* *17, Figure 5a). The analysis of independent contrasts showed that evolutionary shifts in body length have been associated with concomitant shifts in spawning migration distance (*F*_1, 15_ = 5.25, *p *=* *.018, Figure 5b).

Total VN was not correlated with spawning migration distance across species (*r *=* *.14, *p *=* *.30, *n *=* *17, Figure 5c), and evolutionary shifts in spawning migration distance were not accompanied by shifts in total VN (*F*_1, 15_ = 1.03, *p *=* *.16, Figure 5d). The result was similar when data for mean rather than maximum VN were used (*F*_1, 15_ = 0.67, *p *=* *.21).

## 4 Discussion

Can neutral spatial sorting and assortative mating (Shine et al., 2011) associated with long‐distance spawning migration explain evolution of body size and vertebral number in *Anguilla* freshwater eels? To answer this question, we first developed the “neutral” version of the spatial sorting hypothesis by arguing that the evolutionary effects of variation in dispersal capacity and assortative mating might not be independent of but interact with natural selection. In addition to generating assortative mating, variation in dispersal capacity together with spatial and temporal variation in quality of spawning area is likely to influence both reproductive success and survival of spawning migrating individuals (Figure 2), and this might contribute to the evolution of dispersal‐enhancing traits. To provide an empirical example of the rationale, we next performed comparative analyses based on data for 18 species of *Anguilla* eels. Specifically, we examined whether longer spawning migration distances are associated with more elongated bodies and an increased number of vertebrae, under the assumption that greater values in these traits positively influence dispersal capacity. Overall, our results are in line with spatial sorting interacting with natural selection as a contributing driver of body length evolution in eels, but the hypothesized link between migration distance and vertebral number was not supported.

### 4.1 Correlated evolution of migration distance and body size

Results from our analysis of phylogenetic independent contrasts showed that evolutionary shifts in spawning migration distance throughout the phylogeny have been associated with concomitant shifts in body length, indicating that these two traits have evolved in concert. This finding is consistent with the hypothesis that spatial sorting has contributed to the evolution of longer bodies in species with longer spawning migration distances, or resulted in evolution of longer migration distances in species with longer bodies. There is empirical evidence to suggest that larger body size is associated with greater dispersal capacity in various types of organisms (Forsman et al., 2011; Foster, 1964; Hemptinne et al., 2012; Jenkins et al., 2007; Lawlor, 1982; Lomolino, 1984, 1985; McDowall et al., 1994; Roff & Fairbairn, 2001).

In a previous study, based on comparisons of 30 species of ladybird beetles introduced into North America, Hemptinne et al. (2012) report that the two largest species extended their range one order of magnitude faster than the smaller species. We are aware of no other previous tests for associations of body size with dispersal distance using a phylogeny‐based comparative approach.

In addition to favoring swimming capacity, a larger body size confers greater fasting endurance owing to the allometric scaling of the relationships linking fat reserves and metabolic rates to body mass (Forsman, 1996; Schmidt‐Nielsen, 1984). Eels do not eat during the very long and challenging spawning migrations (Tesch, 2003). Being able to survive on stored energy (fat) is therefore of paramount importance for eels (Righton et al., 2016), and this may have contributed to the observed association of body length with migration distance.

Body size is a complex trait associated with other (e.g., physiology and life‐history) phenotypic dimensions (Forsman, 1996, 2015; Forsman & Shine, 1995; Roff, 1992; Schmidt‐Nielsen, 1984). Accordingly, the evolutionary shifts of body size in the *Anguilla* clade indicated by our results have likely been influenced by processes including natural selection operating in addition to and together with “neutral” spatial sorting. As mentioned above, variation among individuals in dispersal capacity and endurance might contribute to differences in reproductive success and survival if the quality of the spawning ground changes in space or through time (Figure 2). The documented evolutionary shifts of body size in *Anguilla* eels might also have been influenced by correlated responses to selection on traits that are developmentally and genetically associated with body size. That we detected an association with migration distance despite this complex nature of body size indicates that the influence of body length on dispersal capacity in eels is strong.

### 4.2 Correlated evolution of abdominal and caudal vertebral numbers

Our results suggested that the abdominal and caudal vertebrae regions have not evolved independently in *Anguilla;* instead, evolutionary shifts of vertebral numbers in these two regions appear to have been correlated. This result is at odds with those of previous studies (Mehta et al., 2010; Ward & Brainerd, 2007). Ward and Brainerd (2007) used data from museum specimens for 54 species, representing seven groups of actinopterygian fishes, together with literature data for additional species representing 14 orders of actinopterygian and elasmobranch fishes. Based on results from analysis of phylogenetic independent contrasts (albeit with branch lengths set to unity), they conclude that in most actinopterygian clades, evolutionary changes in abdominal and caudal VN have not been tightly linked and further suggest that the phylogenetic level of the analyses was too coarse for the level at which VN varies in Elopomorpha. In a more recent study, Mehta et al. (2010) used data for a phylogenetically more homogeneous group consisting of 54 species of elopomorph anguilliform fishes. They report a positive relationship between VN in the caudal and precaudal region (result from analysis where species were treated as independent observations, *r *=* *.42, *n *=* *40, *p *=* *.05), but results from independent contrasts indicate that evolutionary shifts in VN in the two regions have been independent (*p *=* *.41, see Figure 3 in Mehta et al. (2010)). The study by Mehta et al. (2010) included data for 4 *Anguilla* species and several more distantly related taxa. By contrast, our present study is restricted to the *Anguilla* genus. It therefore seems likely that the difference in outcome between these earlier studies (Mehta et al., 2010; Ward & Brainerd, 2007) and our present analysis might be related to the phylogenetic level of resolution used in the analysis. Variation among the different groups of fishes in life‐history, behaviors, habitat use, diets, and lifestyle may also have contributed to differential selection and alternative evolutionary solutions in the different clades.

The association of evolutionary changes of abdominal and caudal vertebral numbers within *Anguilla* indicated by our present results might reflect that there is an optimal combination of VN in these bodily regions that is favored by correlational selection (Arnold, 1988; Arnold & Bennett, 1988). An additional explanation might be that strong genetic correlations and shared developmental pathways promote evolutionary change along the genetic lines of least resistance, while constraining shifts away from the typical relationship (Schluter, 1996).

### 4.3 Independent evolution of vertebral number and migration distance

Our results did not support the hypothesis that evolutionary changes in migration distance have been correlated with evolutionary changes in number of vertebrae in freshwater *Anguilla* eels. This outcome is difficult to reconcile with the large body of evidence pointing to a functional relationship between VN and swimming performance (Ackerly & Ward, 2016; Arnold, 1988; Arnold & Bennett, 1988; Brainerd & Patek, 1998; Kelley et al., 1997; Long et al., 2011; McDowall, 2003; Webb, 1975). A partial explanation for this discrepancy might be that VN also influences aspects of performance and fitness that are not directly related to the capacity for long‐distance dispersal. For instance, it has been proposed that body elongation and VN are related to feeding performance, such as rotational feeding and knotting behavior used by these gape‐limited predators to tear large prey into smaller ingestible pieces (Mehta et al., 2010). Previous studies also report on associations of VN with neonate body size (Arnold & Bennett, 1988; Harding, 1985; Lindell et al., 1993), juvenile growth rate (Swain, 1992a; Tibblin et al., 2016), survival (Lindell et al., 1993; Swain, 1992b; Tibblin et al., 2016), and female reproductive investment (Tibblin et al., 2016). In addition, the distribution of VN may be affected by stabilizing selection operating within populations, as recently reported for pike (Tibblin et al., 2016). This type of variance reducing selection may have countered evolution of greater VN within, and hindered diversification among, the different species.

Phenotypic traits are influenced by the combined effects of genes and developmental plasticity in response to environmental influences (Forsman, 2015; Roff, 1997). Relatively high VN and relatively low VN seem to be associated with exposure to extreme temperature conditions during early embryonic development (Arnold, 1988; Fowler, 1970; Lindsey, 1988). It is perhaps unlikely that temperature conditions at the great water depths at which eels are assumed to reproduce are sufficiently heterogeneous to induce any plasticity of VN (but see Tucker, 1959). However, other environmental stressor(s) that modify gene regulation, activate otherwise unexpressed genes, and impair canalization might impact on VN. It is conceivable that such stressors might induce developmental perturbations in the form of fitness reducing phenotypic abnormalities (Forsman, Merilä, & Lindell, 1994; Hoffman & Parsons, 1991; Merilä, Forsman, & Lindell, 1992) and that these abnormalities may be related to variation in VN. Individuals with unusually high (or low) vertebral counts may therefore be removed from the population by natural selection before they initiate spawning migration or have a chance to reproduce. Moreover, if the highest vertebral counts largely result from plastic responses (i.e., have a nongenetic basis), then those individuals with the most extreme trait values may not contribute to the evolution of the trait even if they do take part in reproduction at the spawning site.

Another, nonbiological, explanation for the lack of association of VN with migration distance is that the number of *Anguilla* species is small. We therefore cannot with certainty discard the possibility that VN has played a role in driving evolution of migration distances (or that migration distance has influenced the evolution of VN) in eels, but that the signature was not detected in our study.

### 4.4 Potential for assortative mating

For neutral spatial sorting to provide a viable contributing explanation for the observed association of body size with migration distance, the dispersal‐enhancing effect of large body size indicated by our present results and previous studies should result in assortative mating (Berggren et al., 2012; Lomolino, 1984; Shine et al., 2011). In the case of, for instance, the European eel *A. anguilla*, assortative mating might happen over a latitudinal gradient in the Sargasso Sea if small individuals with a poor swimming capacity breed further north (closer to Europe) whereas larger and faster swimming individuals breed further south (Figure 2a). Assortative mating according to dispersal capacity (body size) might also happen if larger fast‐dispersing individuals arrive to the spawning area before the smaller individuals (Figure 2b). Assortative interbreeding between fast dispersers (or between slow dispersers) alone will produce offspring having more extreme values for dispersing‐enhancing traits (Berggren et al., 2012; Shine et al., 2011). Unlike the situation at expanding invasion fronts where the sorting is cumulative across generations, any assortative mating potentially resulting from “neutral” spatial or temporal sorting in eels would contribute to increased variance in dispersal‐enhancing traits but without resulting in any evolutionary shift of the mean of the dispersal trait frequency distribution. However, the resulting evolutionary change may be even stronger if the spatial and temporal sorting is combined with traditional natural selection. An association of dispersal capacity with fitness could occur, for instance, if the quality of the spawning area is spatially heterogeneous (Figure 2a) or changes over time (Figure 2b). To our knowledge, the data required to formally evaluate these filtering mechanism hypotheses for eels are not yet available. However, it has been suggested that migration behavior and assortative mating resulting from temporal segregation of reproduction can drive evolutionary change in other organisms (e.g., Bearhop et al., 2005).

### 4.5 Evaluating the role of spatial sorting for evolution of sea snakes

Sea snakes (Elapidae: Hydrophiinae) have many morphological adaptations for life in aquatic environments (Brischoux & Shine, 2011), some of which are shared with eels, suggesting that these disparate lineages might represent an example convergent evolution. Sea snakes also vary in body sizes and movement patterns (Culotta & Pickwell, 1993), and may thus offer an opportunity to further assess the general applicability of the spatial sorting hypothesis by investigating whether selection for migration has influenced the divergence in VN among species and to explore whether independent parallel evolution has occurred in these distinct phylogenetic groups.

## 5 Summary and Conclusions

We propose that the applicability of the spatial sorting hypothesis can be extended from range‐expanding species to a broader range of systems by relaxing the neutral conditions and accepting that neutral sorting may not be independent of but operate together with and increase the effect of natural selection on traits that enhance dispersal capacity and endurance (Figure 2). We use data on spawning migration in *Anguilla* eels as an empirical example of the rationale. The findings from our comparative analyses are consistent with the hypothesis that spatial sorting associated with spawning migration has contributed to evolutionary divergence of body size in freshwater eels. However, our analyses uncovered no association of spawning migration with VN. This might indicate that VN does not contribute to any important degree to swimming performance and long‐distance dispersal capacity of eels. Alternatively, individuals at the tails of the VN frequency distribution might represent suboptimal phenotypes with reduced fitness. It is also possible that extreme VN values represent (nongenetic) developmental plastic responses, such that they are not passed on to the next generations. Previously, studies of microevolutionary change in toads (Phillips et al., 2006; Shine et al., 2011), voles (Forsman et al., 2011), grasshoppers (Berggren et al., 2012), and birds (Berthouly‐Salazar et al., 2012) have been interpreted as support that nonrandom dispersal manifest as spatial sorting can be an important driver of within‐species diversity. Our demonstration of macroevolutionary diversification and correlated shifts in body sizes and spawning migration distances among species of eels within the *Anguilla* clade is important because it adds generality to this issue. It does so by expanding the list of organisms, the ecological settings, and the hierarchical levels of biological organization for which the spatial sorting hypothesis seems to have predictive power.

The novel finding and conclusion from this study, that spatial sorting and long‐distance spawning migration may have influenced the evolution of body size in *Anguilla* eels, will hopefully spur future research, inform policy and practice, and further the understanding of these fascinating, socioeconomically important and endangered animals.

## Conflict of Interest

None declared.

## Data Accessibility

All data used for the analyses are available in Table 1 and Table S1.

## References

[ece32671-bib-0001] Ackerly, K. L. , & Ward, A. B. (2016). How temperature‐induced variation in musculoskeletal anatomy affects escape performance and survival of zebrafish (*Danio rerio*). Journal of Experimental Zoology Part A: Ecological Genetics and Physiology, 325, 25–40. 10.1002/jez.1993 26499994

[ece32671-bib-0002] Amilhat, E. , Aarestrup, K. , Faliex, E. , Simon, G. , Westerberg, H. , & Righton, D. (2016). First evidence of European eels exiting the Mediterranean Sea during their spawning migration. Scientific Reports, 6, 21817. 26906289 10.1038/srep21817 PMC4764813

[ece32671-bib-0003] Aoyama, J. (2009). Life history and evolution of migration in Catadromous eels (Genus *Anguilla*). Aqua‐BioSci Monographs, 2, 1–42.

[ece32671-bib-0004] Aoyama, J. , & Tsukamoto, K. (1997). Evolution of the freshwater eels. Naturwissenschaften, 84, 17–21. 9050003 10.1007/s001140050340

[ece32671-bib-0005] Aoyama, J. , Wouthuyzen, S. , Miller, M. J. , Inagaki, T. , & Tsukamoto, K. (2003). Short‐distance spawning migration of tropical freshwater eels. Biological Bulletin, 204, 104–108. 12588749 10.2307/1543500

[ece32671-bib-0006] Arnold, S. J. (1988). Quantitative genetics and selection in natural populations: Microevolution of vertebral numbers in the garter snake *Thamnophis elegans* In Weir B. S., Goodman M. M., Eisen E. J., & Namkong G. (Eds.), Proceedings of the second international conference on quantitative genetics (pp. 619–636). Sunderland, Massachusetts: Sinauer Associates.

[ece32671-bib-0007] Arnold, S. J. , & Bennett, A. F. (1988). Behavioral variation in natural populations. V. Morphological correlates of locomotion in the garter snake *Thamnophis radix* . Biological Journal of the Linnean Society, 34, 175–190.

[ece32671-bib-0008] Bearhop, S. , Fiedler, W. , Furness, R. W. , Votier, S. C. , Waldron, S. , Newton, J. , … Farnsworth, K. (2005). Assortative mating as a mechanism for rapid evolution of a migratory divide. Science, 310, 502–504. 16239479 10.1126/science.1115661

[ece32671-bib-0009] Beguer‐Pon, M. , Castonguay, M. , Shan, S. , Benchetrit, J. , & Dodson, J. J. (2015). Direct observations of American eels migrating across the continental shelf to the Sargasso Sea. Nature Communications, 6, 8705. 10.1038/ncomms9705 PMC4918406 26505325

[ece32671-bib-0010] Berggren, H. , Tinnert, J. , & Forsman, A. (2012). Spatial sorting may explain evolutionary dynamics of wing polymorphism in pygmy grasshoppers. Journal of Evolutionary Biology, 25, 2126–2138. doi: 2110/1111/j.1420‐9101.2012.02592.x 22901281 10.1111/j.1420-9101.2012.02592.x

[ece32671-bib-0011] Berthouly‐Salazar, C. , van Rensburg, B. J. , Le Roux, J. J. , van Vuuren, B. J. , & Hui, C. (2012). Spatial sorting drives morphological variation in the invasive bird, *Acridotheris tristis* . PLoS ONE, 7, e38145. 22693591 10.1371/journal.pone.0038145 PMC3364963

[ece32671-bib-0012] Bolnick, D. I. , & Otto, S. P. (2013). The magnitude of local adaptation under genotype‐dependent dispersal. Ecology and Evolution, 3, 4722–4735. 24363900 10.1002/ece3.850 PMC3867907

[ece32671-bib-0013] Brainerd, E. L. , & Patek, S. N. (1998). Vertebral column morphology, C‐start curvature, and the evolution of mechanical defenses in tetraodontiform fishes. Copeia, 1998, 971–984.

[ece32671-bib-0014] Brischoux, F. , & Shine, R. (2011). Morphological adaptations to marine life in snakes. Journal of Morphology, 272, 566–572. 21337377 10.1002/jmor.10933

[ece32671-bib-0015] Culotta, W. A. , & Pickwell, G. V. (1993). The Venomous sea snakes: A comprehensive bibliography. Malabar, FL: Krieger Publishing Company.

[ece32671-bib-0016] Edelaar, P. , & Bolnick, D. I. (2012). Non‐random gene flow: An underappreciated force in evolution and ecology. Trends in Ecology & Evolution, 27, 659–665. 22884295 10.1016/j.tree.2012.07.009

[ece32671-bib-0017] Edelaar, P. , Siepielski, A. M. , & Clobert, J. (2008). Perspective – Matching habitat choice causes directed gene flow: A neglected dimension in evolution and ecology. Evolution, 62, 2462–2472. 18637835 10.1111/j.1558-5646.2008.00459.x

[ece32671-bib-0018] Ege, V. (1939). A revision of the genus Anguilla Shaw: A systematic, phylogenetic and geographical study. Dana‐Report, 16, 1–256.

[ece32671-bib-0019] Felsenstein, J. (1985). Phylogenies and the comparative method. The American Naturalist, 125, 1–15. 10.1086/703055 31094602

[ece32671-bib-0020] FishBase (2015). FishBase. World Wide Web electronic publication. (eds R. Froese & D. Pauly). www.fishbase.org.

[ece32671-bib-0021] Forsman, A. (1996). Body size and net energy gain in gape‐limited predators: A model. Journal of Herpetology, 30, 307–319.

[ece32671-bib-0022] Forsman, A. (2015). Rethinking phenotypic plasticity and its consequences for individuals, populations and species. Heredity, 115, 276–284. 25293873 10.1038/hdy.2014.92 PMC4815454

[ece32671-bib-0023] Forsman, A. , Merilä, J. , & Ebenhard, T. (2011). Phenotypic evolution of dispersal‐enhancing traits in insular voles. Proceedings of the Royal Society B‐Biological Sciences, 278, 225–232. 10.1098/rspb.2010.1325 PMC3013397 20685710

[ece32671-bib-0024] Forsman, A. , Merilä, J. , & Lindell, L. E. (1994). Do scale anomalies cause differential survival in *Vipera berus* . Journal of Herpetology, 28, 435–440.

[ece32671-bib-0025] Forsman, A. , & Shine, R. (1995). Sexual size dimorphism in relation to frequency of reproduction in turtles (Testudines: Emydidae). Copeia, 1995, 727–729.

[ece32671-bib-0026] Foster, J. B. (1964). Evolution of mammals on islands. Nature, 202, 234–235.

[ece32671-bib-0027] Fowler, J. A. (1970). Control of vertebral number in teleosts – An embryological problem. Quarterly Review of Biology, 45, 148.

[ece32671-bib-0028] Garland, T. , Harvey, P. H. , & Ives, A. R. (1992). Procedures for the analysis of comparative data using phylogenetically independent contrasts. Systematic Biology, 41, 18–32.

[ece32671-bib-0029] Harding, E. F. (1985). On the homogeneity of the european eel population (*Anguilla anguilla*). Dana‐a Journal of Fisheries and Marine Research, 4, 49–66.

[ece32671-bib-0030] Harvey, P. H. , & Pagel, M. D. (1991). The comparative method in evolutionary biology. Oxford, United Kingdom: Oxford University Press.

[ece32671-bib-0031] Hemptinne, J. L. , Magro, A. , Evans, E. W. , & Dixon, A. F. G. (2012). Body size and the rate of spread of invasive ladybird beetles in North America. Biological Invasions, 14, 595–605.

[ece32671-bib-0032] Hoffman, A. A. , & Parsons, P. A. (1991). Evolutionary genetics and environmental stress. New York, NY: Oxford University Press.

[ece32671-bib-0033] IUCN (2015) The IUCN Red List of Threatened Species. Version 2015.4.

[ece32671-bib-0034] Jenkins, D. G. , Brescacin, C. R. , Duxbury, C. V. , Elliott, J. A. , Evans, J. A. , Grablow, K. R. , … Williams, S. E. (2007). Does size matter for dispersal distance? Global Ecology and Biogeography, 16, 415–425.

[ece32671-bib-0035] Kelley, K. C. , Arnold, S. J. , & Glatstone, J. (1997). The effects of substrate and vertebral number on locomotion in the garter snake *Thamnophis elegans* . Functional Ecology, 11, 189–198.

[ece32671-bib-0036] Lawlor, T. E. (1982). The evolution of body size in mammals – Evidence from insular populations in Mexico. The American Naturalist, 119, 54–72.

[ece32671-bib-0037] Lindell, L. E. (1994). The evolution of vertebral number and body size in snakes. Functional Ecology, 8, 708–719.

[ece32671-bib-0038] Lindell, L. E. , Forsman, A. , & Merilä, J. (1993). Variation in number of ventral scales in snakes – Effects on body size, growth‐rate and survival in the adder, *Vipera berus* . Journal of Zoology, 230, 101–115.

[ece32671-bib-0039] Lindsey, C. C. (1975). Pleomerism, the widespread tendency among related fish species for vertebral number to be correlated with maximum body length. Journal of the Fisheries Research Board of Canada, 32, 2453–2469.

[ece32671-bib-0040] Lindsey, C. C. (1988). Factors controlling meristic variation In Hoar D. S., & Randall D. J. (Eds.), Fish physiology (pp. 197–204). London: Academic Press.

[ece32671-bib-0041] Lomolino, M. V. (1984). Immigrant selection, predation, and the distributions of *Microtus pennsylvanicus* and *Blarina brevicauda* on islands. The American Naturalist, 123, 468–483.

[ece32671-bib-0042] Lomolino, M. V. (1985). Body size of mammals on islands – The island rule reexamined. The American Naturalist, 125, 310–316.

[ece32671-bib-0043] Long, J. H. , Krenitsky, N. M. , Roberts, S. F. , Hirokawa, J. , de Leeuw, J. , & Porter, M. E. (2011). Testing biomimetic structures in bioinspired robots: How vertebrae control the stiffness of the body and the behavior of fish‐like swimmers. Integrative and Comparative Biology, 51, 158–175. 21576117 10.1093/icb/icr020

[ece32671-bib-0044] Maxwell, E. , & Wilson, L. (2013). Regionalization of the axial skeleton in the ‘ambush predator’ guild ‐ are there developmental rules underlying body shape evolution in ray‐finned fishes? BMC Evolutionary Biology, 13, 265. 24314064 10.1186/1471-2148-13-265 PMC3867419

[ece32671-bib-0045] McDowall, M. (2003). Variation in vertebral number in galaxiid fishes, how fishes swim and a possible reason for pleomerism. Reviews in Fish Biology and Fisheries, 13, 247–263.

[ece32671-bib-0046] McDowall, R. M. , Mitchell, C. P. , & Brothers, E. B. (1994). Age at migration from the sea of juvenile Galaxias in New Zealand (Pisces, Galaxiidae). Bulletin of Marine Sciences, 54, 385–402.

[ece32671-bib-0047] Mehta, R. S. , Ward, A. B. , Alfaro, M. E. , & Wainwright, P. C. (2010). Elongation of the body in eels. Integrative and Comparative Biology, 50, 1091–1105. 21558261 10.1093/icb/icq075

[ece32671-bib-0048] Merilä, J. , Forsman, A. , & Lindell, L. E. (1992). High‐frequency of ventral scale anomalies in *Vipera berus* populations. Copeia, 1992, 1127–1130.

[ece32671-bib-0049] Minegishi, Y. , Aoyama, J. , Inoue, J. G. , Miya, M. , Nishida, M. , & Tsukamoto, K. (2005). Molecular phylogeny and evolution of the freshwater eels genus *Anguilla* based on the whole mitochondrial genome sequences. Molecular Phylogenetics and Evolution, 34, 134–146. 15579387 10.1016/j.ympev.2004.09.003

[ece32671-bib-0050] Phillips, B. L. , Brown, G. P. , Webb, J. K. , & Shine, R. (2006). Invasion and the evolution of speed in toads. Nature, 439, 803. 16482148 10.1038/439803a

[ece32671-bib-0051] Purvis, A. , & Rambaut, A. (1995). Comparative analysis by independent contrasts (C.A.I.C.): An Apple Macintosh application for analysing comparative data. Computer Applied Biosciences, 11, 247–251. 10.1093/bioinformatics/11.3.247 7583692

[ece32671-bib-0052] Reznick, D. N. , & Ghalambor, C. K. (2001). The population ecology of contemporary adaptations: What empirical studies reveal about the conditions that promote adaptive evolution. Genetica, 112–113, 183–198. 11838765

[ece32671-bib-0053] Rice, W. R. , & Gaines, S. D. (1994). Extending nondirectional heterogeneity tests to evaluate simply ordered alternative hypotheses. Proceedings of the National Academy of Sciences of the United States of America, 91, 225–226. 8278369 10.1073/pnas.91.1.225 PMC42919

[ece32671-bib-0054] Righton, D. , Westerberg, H. , Feunteun, E. , Økland, F. , Gargan, P. , Amilhat, E. , … Aarestrup, K. (2016). Empirical observations of the spawning migration of European eels: The long and dangerous road to the Sargasso Sea. Science Advances, 2, e1501694. 27713924 10.1126/sciadv.1501694 PMC5052013

[ece32671-bib-0055] Roff, D. A. (1992). The evolution of life histories: Theory and analysis. New York, NY: Chapman & Hall Inc..

[ece32671-bib-0056] Roff, D. A. (1997). Evolutionary quantitative genetics. New York, NY: Chapman & Hall.

[ece32671-bib-0057] Roff, D. A. , & Fairbairn, D. J. (2001). The genetic basis of dispersal and migration, and its consequences for the evolution of correlated traits In Clobert J., Danchin E., Dhondt A. A., & Nichols J. D. (Eds.), Dispersal (pp. 191–202). New York, NY: Oxford University Press.

[ece32671-bib-0058] Schluter, D. (1996). Adaptive radiation along genetic lines of least resistance. Evolution, 50, 1766–1774. 10.1111/j.1558-5646.1996.tb03563.x 28565589

[ece32671-bib-0059] Schmidt‐Nielsen, K. (1984). Scaling: Why is animal size so important?. Cambridge: Cambridge University Press.

[ece32671-bib-0060] Shine, R. , Brown, G. P. , & Phillips, B. L. (2011). An evolutionary process that assembles phenotypes through space rather than through time. Proceedings of the National Academy of Sciences of the United States of America, 108, 5708–5711. 21436040 10.1073/pnas.1018989108 PMC3078378

[ece32671-bib-0061] Silfvergrip, A. M. C. (2009). CITES identification guide to the freshwater eels (Anguillidae) – With focus on the European eel Anguilla anguilla. Stockholm: Swedish Environmental Protection Agency.

[ece32671-bib-0062] Swain, D. P. (1992a). The functional basis of natural‐selection for vertebral traits of larvae in the stickleback *Gasterosteus aculeatus* . Evolution, 46, 987–997. 10.1111/j.1558-5646.1992.tb00614.x 28564394

[ece32671-bib-0063] Swain, D. P. (1992b). Selective predation for vertebral phenotype in *Gasterosteus aculeatus* ‐ reversal in the direction of selection at different larval sizes. Evolution, 46, 998–1013. 10.1111/j.1558-5646.1992.tb00615.x 28564417

[ece32671-bib-0064] Tesch, F. W. (2003). The eel, 5th ed Oxford, United Kingdom: Blackwell Sciences.

[ece32671-bib-0065] Tibblin, P. , Berggren, H. , Nordahl, O. , Larsson, P. , & Forsman, A. (2016). Causes and consequences of intra‐specific variation in vertebral number. Scientific Reports, 6, 26372. 27210072 10.1038/srep26372 PMC4876516

[ece32671-bib-0066] Tsukamoto, K. , Chow, S. , Otake, T. , Kurogi, H. , Mochioka, N. , Miller, M. J. , … Tanaka, H. (2011). Oceanic spawning ecology of freshwater eels in the western North Pacific. Nature Communications, 2, 179. 10.1038/ncomms1174 PMC3105336 21285957

[ece32671-bib-0067] Tucker, D. W. (1959). A new solution to the Atlantic eel problem. Nature, 183, 495–501. 10.1038/1831405a0 13657143

[ece32671-bib-0068] Ward, A. B. , & Brainerd, E. L. (2007). Evolution of axial patterning in elongate fishes. Biological Journal of the Linnean Society, 90, 97–116.

[ece32671-bib-0069] Webb, P. W. (1975). Hydrodynamics and energetics of fish propulsion. Bulletin of the Fisheries Research Board of Canada, 190, 1–156.

