# Supplementary material for: Can spatial sorting associated with spawning migration explain evolution of body size and vertebral number in Anguilla eels?
Source: Ecol Evol. 2016 Dec 25;7(2):751–61. doi: 10.1002/ece3.2671 (PMC5243785; doi:10.1002/ece3.2671)
Supplement: Supplementary file 1 [file ECE3-7-751-s001.docx]

| **Species** |  | **Presumed spawning site** | **Approximate latitudinal range^1^** | **Approximate longitudinal range^1^** | **Endmost distribution point^1^** | **Distance to spawning area (km)** |
| --- | --- | --- | --- | --- | --- | --- |
| *A. anguilla* |  | Sargasso Sea. 26°N, 60°W^1^ | 22°N-72°N | 22°W-45°E | 67°N, 41°E | 8200 |
| *A. australis australis* |  | West of South Equatorial current. 18°S, 170°W^1, 2^ | 45°S-36°S | 140°E-162°E | 37°S, 140°E | 5500 |
| *A. bengalensis bengalensis* |  | Southwest of Sumatra. 5.5°S, 97°E^1^ | 2°N-30°N | 70°E-98°E | 21°N, 73°E | 4400 |
| *A. bicolor bicolor* |  | Southwest of Sumatra. 5.5°S, 97°E^1^ | 24°S-25°N | 71°E-124°E | 18°S, 120°E | 3000 |
| *A. borneensis* |  | Northwest of New Guinea: Celebes Sea. 3°N, 122°E^1, 3^ | 2°S-2°N | 115°E-117°E | 2°S, 115°E | 750 |
| *A. celebesensis* |  | Northwest of New Guinea: Tomini Bay. 1°S, 121°E^1, 3^ | 14°S-17°N | 99°E-144°E | 0°S/N, 99°E | 4400 |
| *A. dieffenbachii* |  | West of South Equatorial current. 18°S, 170°W^1, 2^ | 47°S-34°S | 166°E-174°W | 47°S, 167°E | 4000 |
| *A. interioris* |  | Northwest of New Guinea: Celebes Sea. 3°N, 122°E^3^ | 11°S-2°S | 142°E-151°E | 10°S-151°E | 3700 |
| *A. japonica* |  | West of Mariana ridge. 14°N, 142°E^1, 4^ | 18°N-43°N | 105°E-143°E | 42°N, 120°E | 3900 |
| *A. bengalensis labiata* |  | East of Madagascar. 15°S, 58°E^1^ | 28°S-10°N | 25°E-50°E | 18°S, 25°E | 4200 |
| *A. marmorata* |  | South Equatorial current. 7°S, 160°W^1, 2^ | 22°S-35°N | 72°E-140°W | 2°S, 128°E | 7400 |
| *A. megastoma* |  | South Equatorial current. 7°S, 160°W^1, 2^ | 25°S-7°S | 155°E-130°W | 8°S, 155°E | 5000 |
| *A. mossambica* |  | East of Madagascar. 15°S, 58°E^1^ | 34°S-1°S | 19°E-50°E | 34°S, 19°E | 4600 |
| *A. obscura* |  | South Equatorial current. 7°S, 160°W^1, 2^ | 30°S-3°N | 128°E-146°W | 2°S, 129°E | 7900 |
| *A. bicolor pacifica* |  | Southwest of Sumatra^1^/Equatorial Pacific region?^2^ | 9°S-15°N | 115°E-153°E | - | - |
| *A. reinhardtii* |  | West of South Equatorial current. 18°S, 170°W^1, 2^ | 36°S-21°S | 145°E-164°E | 36°S, 149°E | 4500 |
| *A. rostrata* |  | Sargasso Sea. 26°N, 60°W^1^ | 5°N-65°N | 42°W-105°W | 65°N, 52°W | 4500 |
| *A. australis schmidtii* |  | West of South Equatorial current. 18°S, 170°W^1, 2^ | 49°S-15°S | 166°E-138°W | 47°S, 167°E | 4000 |

**Table S1.** Eel species of the genus *Anguilla*; the presumed spawning area, distribution area in both latitudinal and longitudinal direction, and spawning migration distance calculated from endmost point in the distribution area (longest possible distance for the individual species).

References; ^1^[Tesch [1]](#_ENREF_1); ^2^[Aoyama [2]](#_ENREF_2); ^3^[Aoyama *et al.* [3]](#_ENREF_3); ^4^[Tsukamoto *et al.* [4]](#_ENREF_4).

1. Tesch F.W. 2003 *The Eel*. 5th ed. Oxford, United Kingdom, Blackwell Sciences; 408 p.

2. Aoyama J. 2009 Life History and Evolution of Migration in Catadromous Eels (Genus *Anguilla*). *Aqua-BioSci Monographs* **2**, 1-42.

3. Aoyama J., Wouthuyzen S., Miller M.J., Inagaki T., Tsukamoto K. 2003 Short-distance spawning migration of tropical freshwater eels. *Biological Bulletin* **204**, 104-108. (doi:10.2307/1543500).

4. Tsukamoto K., Chow S., Otake T., Kurogi H., Mochioka N., Miller M.J., Aoyama J., Kimura S., Watanabe S., Yoshinaga T., et al. 2011 Oceanic spawning ecology of freshwater eels in the western North Pacific. *Nature Communications* **2**, 179. (doi:<http://www.nature.com/ncomms/journal/v2/n2/suppinfo/ncomms1174_S1.html>).
